# Nomogram for Predicting the Postoperative Venous Thromboembolism in Spinal Metastasis Tumor: A Multicenter Retrospective Study

**DOI:** 10.3389/fonc.2021.629823

**Published:** 2021-06-24

**Authors:** Hao-ran Zhang, Ming-you Xu, Xiong-gang Yang, Feng Wang, Hao Zhang, Li Yang, Rui-qi Qiao, Ji-kai Li, Yun-long Zhao, Jing-yu Zhang, Yong-cheng Hu

**Affiliations:** ^1^ Department of Bone Tumor, Tianjin Hospital, Tianjin, China; ^2^ Department of Orthopedics, Huashan Hospital, Fudan University, Shanghai, China

**Keywords:** venous thromboembolism, deep vein thrombosis, pulmonary embolism, spinal metastasis, prediction model

## Abstract

**Introduction:**

Venous thromboembolism can be divided into deep vein thrombosis and pulmonary embolism. These diseases are a major factor affecting the clinical prognosis of patients and can lead to the death of these patients. Unfortunately, the literature on the risk factors of venous thromboembolism after surgery for spine metastatic bone lesions are rare, and no predictive model has been established.

**Methods:**

We retrospectively analyzed 411 cancer patients who underwent metastatic spinal tumor surgery at our institution between 2009 and 2019. The outcome variable of the current study is venous thromboembolism that occurred within 90 days of surgery. In order to identify the risk factors for venous thromboembolism, a univariate logistic regression analysis was performed first, and then variables significant at the P value less than 0.2 were included in a multivariate logistic regression analysis. Finally, a nomogram model was established using the independent risk factors.

**Results:**

In the multivariate logistic regression model, four independent risk factors for venous thromboembolism were further screened out, including preoperative Frankel score (OR=2.68, 95% CI 1.78-4.04, P=0.001), blood transfusion (OR=3.11, 95% CI 1.61-6.02, P=0.041), Charlson comorbidity index (OR=2.01, 95% CI 1.27-3.17, P=0.013; OR=2.29, 95% CI 1.25-4.20, P=0.017), and operative time (OR=1.36, 95% CI 1.14-1.63, P=0.001). On the basis of the four independent influencing factors screened out by multivariate logistic regression model, a nomogram prediction model was established. Both training sample and validation sample showed that the predicted probability of the nomogram had a strong correlation with the actual situation.

**Conclusion:**

The prediction model for postoperative VTE developed by our team provides clinicians with a simple method that can be used to calculate the VTE risk of patients at the bedside, and can help clinicians make evidence-based judgments on when to use intervention measures. In clinical practice, the simplicity of this predictive model has great practical value.

## Introduction

Venous thromboembolism (VTE) can be divided into deep vein thrombosis (DVT) and pulmonary embolism (PE). These diseases are a major factor affecting the clinical prognosis of patients and can lead to the death of these patients. Unfortunately, cancer patients have a higher risk of VTE than other patients (1, 2). In addition, spinal surgery is also considered an independent risk factor for VTE (3, 4). Patients with spinal metastases have both the characteristics of cancer patients and the need for spinal surgery. Therefore, it is reasonable to believe that this patient population has a higher prevalence of VTE. A recent retrospective study showed that 11% of patients with spinal metastases undergoing spinal surgery were observed to have symptomatic VTE (5). Accurately identifying risk factors related to VTE can help clinicians and patients determine which high-risk groups can be treated with interventions as soon as possible, which is very helpful for reducing perioperative mortality and improving postoperative survival time and quality of life.

For the risk factors of VTE after spinal surgery, some studies have reported the corresponding results, and some risk scores have been established (6–8). However, it is still unclear whether these conclusions are equally applicable to spinal metastasis surgery, because the treatment measures and prognostic characteristics of patients with spinal metastases are very specific (5). Therefore, it is very necessary to identify the risk factors of VTE in patients with spinal metastases. Unfortunately, the literature on the risk factors of VTE after surgery for spine metastatic bone lesions are rare (5, 9), and no predictive model has been established. Groot et al. found that longer duration of surgery was independently associated with an increased risk of symptomatic VTE (5). Kaewborisutsakul et al. found that patients who underwent surgery for extramedullary spinal tumors showed a 2.9% incidence of DVT and risk factors associated with DVT occurrence were operative time ≥8 h and plasma transfusion (9).

Nomogram model has been widely used in prognostic research and risk assessment of cancer patients (10–12). This prediction method transforms the traditional regression model into a visual risk assessment for each patient by creating a user-friendly graph, which is undoubtedly convenient and accurate. And compared with the traditional scoring table, the nomogram has proven to be more reliable than other systems, so it has been suggested as an alternative or even a new standard (13). Through the nomogram model, clinicians can show patients their predictions of future events more vividly, instead of roughly reporting corresponding risk factors, which also has a positive effect on improving patient compliance.

Therefore, in this study, we try to determine the risk factors related to VTE in patients undergoing spinal metastasis surgery and establish a nomogram prediction model.

## Patients and Methods

### Participants

We retrospectively analyzed 411 cancer patients who underwent metastatic spinal tumor surgery at our institution between October 2009 and April 2019. The indications for surgery were worsening neurological function, existing or potential spinal instability, pain that cannot be alleviated, or a combination of these factors. The exclusion criteria were as follows: minimally invasive surgery for spinal metastases, revision procedures, a VTE within 2 weeks before surgery, patients with coagulopathy, and surgery for sacral metastases. This study received ethical approval from the institutional review board and each patient obtained informed consent.

### Description of Study Population

Among all patients, 230 (56.0%) were male patients and 181 (44.0%) were female patients. 49 (11.9%) patients had tumors in the cervical region, 206 (50.1%) patients had tumors in the thoracic region, and 156 (38.0%) patients had tumors in the lumbar area. 250 (60.8%) patients had more than one spinal metastasis. 246 (59.9%) patients and 239 (58.2%) patients received preoperative radiotherapy and chemotherapy, respectively. 302 (73.5%) patients were able to walk with or without aids before surgery. In order to better validate the model, we divided the entire study population into training sample and validation sample, and the two samples maintained similarity between various indicators (**Table 1**).

**Table 1:** Baseline characteristics of the study population.

Characteristics	All patients	Training sample	Validation sample	P value
Number	411	288	123	
Gender, N (%)				0.492
male	230 (56.0%)	158 (54.9%)	72 (58.5%)	
female	181 (44.0%)	130 (45.1%)	51 (41.5%)	
Age, mean ± SD	58.4 ± 10.6	58.8 ± 10.2	57.4 ± 11.4	0.239
Type of tumor, N (%)				0.667
rapid	191 (46.5%)	138 (47.9%)	53 (43.1%)	
moderate	164 (39.9%)	112 (38.9%)	52 (42.3%)	
slow	56 (13.6%)	38 (13.2%)	18 (14.6%)	
Tumor location, N (%)				0.906
cervical	49 (11.9%)	33 (11.5%)	16 (13.0%)	
thoracic	206 (50.1%)	145 (50.3%)	61 (49.6%)	
lumbar	156 (38.0%)	110 (38.2%)	46 (37.4%)	
Number of spinal metastases, N (%)				0.356
single	161 (39.2%)	117 (40.6%)	44 (35.8%)	
multiple	250 (60.8%)	171 (59.4%)	79 (64.2%)	
BMI (kg/m^2^), N (%)				0.991
< 18.5	13 (3.2%)	9 (3.1%)	4 (3.3%)	
18.5-30	349 (84.9%)	245 (85.1%)	104 (84.6%)	
> 30	49 (11.9%)	34 (11.8%)	15 (12.2%)	
Surgical procedure, N (%)				0.351
type 1	68 (16.5%)	43 (14.9%)	25 (20.3%)	
type 2	319 (77.6%)	229 (79.5%)	90 (73.2%)	
type 3	24 (5.8%)	16 (5.6%)	8 (6.5%)	
Preoperative radiotherapy, N (%)				0.426
yes	246 (59.9%)	176 (61.1%)	70 (56.9%)	
no	165 (40.1%)	112 (38.9%)	53 (43.1%)	
Preoperative chemotherapy, N (%)				0.441
yes	239 (58.2%)	171 (59.4%)	68 (55.3%)	
no	172 (41.8%)	117 (40.6%)	55 (44.7%)	
Visceral metastases, N (%)				0.462
no	100 (24.3%)	73 (25.3%)	27 (22.0%)	
yes	311 (75.7%)	215 (74.7%)	96 (78.0%)	
Blood loss (liters), mean ± SD	1.3 ± 1.1	1.2 ± 0.9	1.3 ± 1.3	0.497
Preoperative Frankel score, N (%)				0.693
A-C	109 (26.5%)	78 (27.1%)	31 (25.2%)	
D-E	302 (73.5%)	210 (72.9%)	92 (74.8%)	
Blood transfusion, N (%)				0.194
yes	177 (43.1%)	130 (45.1%)	47 (38.2%)	
no	234 (56.9%)	158 (54.9%)	76 (61.8%)	
Charlson comorbidity index, N (%)				0.131
6	63 (15.3%)	42 (14.6%)	21 (17.1%)	
7	117 (28.5%)	75 (26.0%)	42 (34.1%)	
≥8	231 (56.2%)	171 (59.4%)	60 (48.8%)	
Operative time (hours), mean ± SD	4.0 ± 1.4	4.0 ± 1.3	4.1 ± 1.6	0.759

### Outcome and Variables

This study was completed according to the “Transparent Reporting of a multivariable prediction model for Individual Prognosis or Diagnosis” statement (14). The outcome variable of the current study is PE or DVT that occurred within 90 days of surgery. The patient presented with calf swelling or tenderness, acute dyspnea, deoxygenation or unexplained shock. DVT is diagnosed by leg ultrasonography, and PE is diagnosed by pulmonary angiography or chest CT in patients with symptoms of pulmonary embolism.

The recorded data included demographic characteristics, primary tumor type, tumor location, number of spinal metastases, BMI, surgical procedure, preoperative radiotherapy, preoperative chemotherapy, visceral metastases, blood loss, preoperative Frankel score, blood transfusion, Charlson comorbidity index, and operative time.

Primary tumor type was divided into 3 groups according to Tomita and colleagues, including rapid group (lung and stomach), moderate group (kidney, liver, uterus, unidentified, and others) and slow group (thyroid, prostate, breast, and rectum) (15). Tumor location included cervical spine, thoracic spine and lumbar spine. The number of spinal metastases was divided into single spinal metastases and multiple spinal metastases. The surgical methods we used varied according to the location and size of metastatic tumors, and can be divided into three categories in general: palliative instrumentation and decompression (type 1), subtotal corpectomy (type 2), and total en bloc spondylectomy (type 3) (10). Reconstruction and stabilization procedures were performed *via* pedicle screws, titanium mesh, bone cement, and bone graft fusion alone or with various combinations. The intraoperative blood loss was obtained from the anesthetist’s medical records and records of intraoperative fluid management. The neurological status of cancer patient before surgery was evaluated according to the Frankel score: patients with A-C grade were considered to be nonambulatory, and patients with D-E grade retained walking function (16). The comorbidity was measured and calculated according to the modified Charlson Comorbidity Index (17).

### Statistical Analysis

Using a computer program, the study population was randomly divided into training sample and validation sample, with a ratio of 7:3. Continuous variables were described as mean ± standard deviation, and categorical variables were described as proportions. The Student’s t-tests (continuous variables) and chi-square tests (categorical variables) were used to confirm any statistical differences between means and proportions.

In order to identify the risk factors for VTE, a univariate logistic regression analysis was performed first, and then variables significant at the P value less than 0.2 were included in a multivariate logistic regression analysis to screen for independent risk factors. A forest plot was used to visualize the results of univariate and multivariate regression analyses. Finally, a nomogram model was established using the independent risk factors screened out by multivariate logistic regression.

Discrimination of the prediction model was validated using the area under the curve (AUC) and the consistence was validated using the calibration curves. The calibration curve plot is a curve fitting graph of the actual occurrence rate and the predicted occurrence rate. The calibration curve is the fitting line between the predicted and actual incidences, and y=x means that the predicted and actual incidences are exactly the same. The closer the two lines are, the closer the predicted and actual occurrence rates are, which further shows that the consistence of the model is better. The prediction model was established and validated according to the study published by Iasonos and colleagues (18). Statistical analysis was performed using R version 3.5.2 for Windows (R Foundation for Statistical Computing, Vienna, Austria), GraphPad Prism 8 Software (GraphPad Software Inc., San Diego, CA), and SPSS 22.0 software (SPSS Inc., Chicago, Illinois, USA). P ≤ 0.05 (two-sided) was considered statistically significant.

## Results

### Clinical Status

There were 49 patients (11.9%) diagnosed with VTE within 90 days after spinal metastasis surgery, of which 42 patients (10.2%) had DVT and seven patients (1.7%) had PE. The mean age of 49 patients was 58.5 years, and there were 20 female patients (40.8%). Among the seven patients with PE, five patients were observed to be accompanied by DVT, and one patient died of PE. For patients with VTE, the mean intraoperative blood loss was 1.4 ± 0.9 liters, the mean operative time was 4.6 ± 0.8 hours, and 33 patients (67.3%) received blood transfusion.

### Risk Factors Associated With VTE

**Figure 1:**
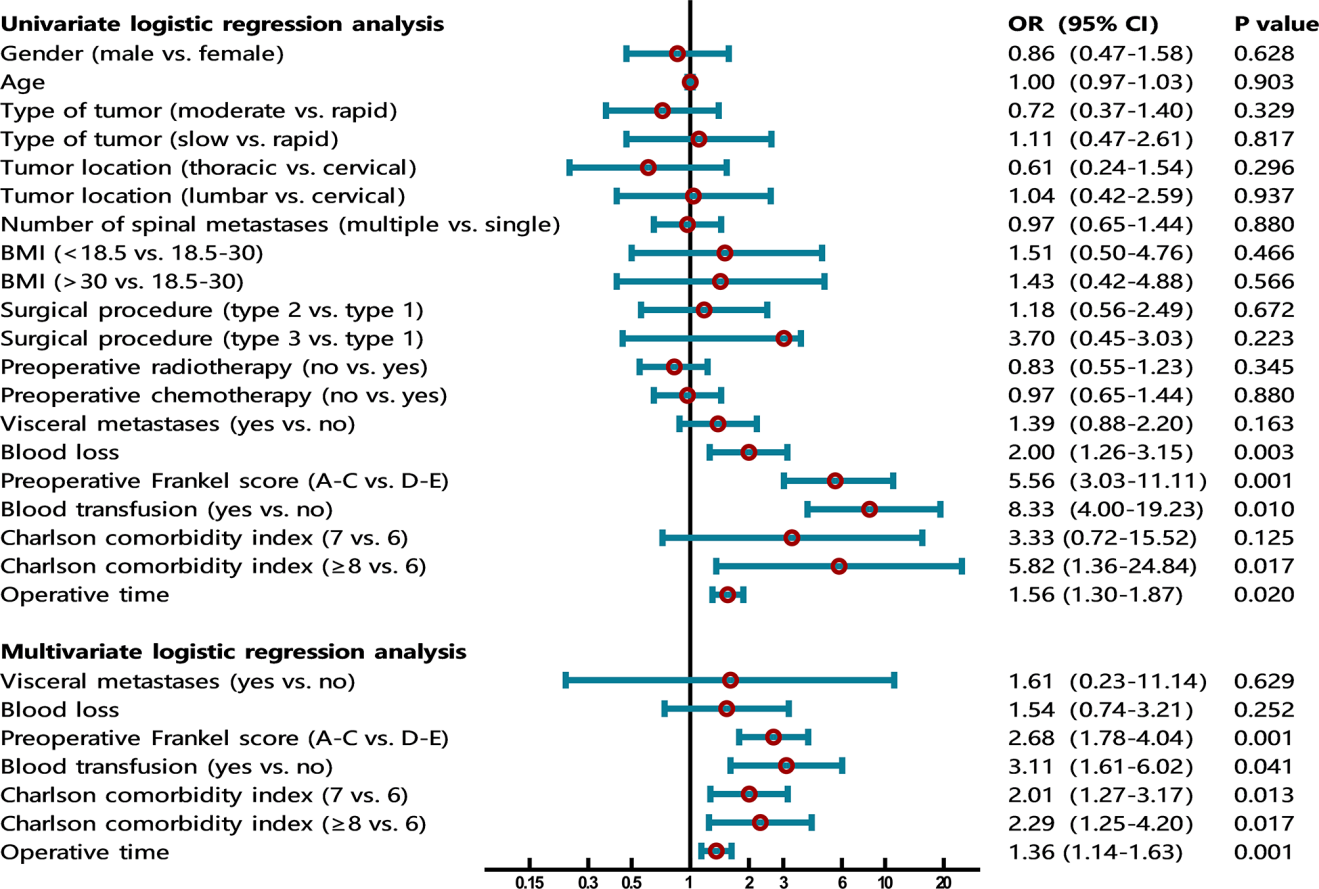
The forest plot shows the results of univariate and multivariate analyses. In the multivariate logistic regression model, four independent risk factors for VTE were further screened out, including preoperative Frankel score (OR=2.68, 95% CI 1.78-4.04, P=0.001), blood transfusion (OR=3.11, 95% CI 1.61-6.02, P=0.041), Charlson comorbidity index (OR=2.01, 95% CI 1.27-3.17, P=0.013; OR=2.29, 95% CI 1.25-4.20, P=0.017), and operative time (OR=1.36, 95% CI 1.14-1.63, P=0.001).

Univariate logistic regression analysis showed that gender (P=0.628), age (P=0.903), primary tumor type (P=0.329, P=0.817), tumor location (P=0.296, P=0.937), number of spinal metastases (P=0.880), BMI (P=0.466, P=0.566), surgical procedure (P=0.672, P=0.223), preoperative radiotherapy (P=0.345), and preoperative chemotherapy (P=0.880) were not statistically significant. These factors did not enter the next statistical test. According to the test level we set, a total of six factors were put into the multivariate logistic regression model, including visceral metastases, blood loss, preoperative Frankel score, blood transfusion, Charlson comorbidity index, and operative time (**Table 2** and **Figure 1**).

**Table 2:** Logistic regression assessing risk factors for venous thromboembolism.

Factor	Univariable analysis			Multivariable analysis		
	OR	95% CI	P value	OR	95% CI	P value
Gender						NI
male	0.86	0.47-1.58	0.628			
female	Ref	Ref	Ref			
Age	1.00	0.97-1.03	0.903			NI
Type of tumor						NI
rapid	Ref	Ref	Ref			
moderate	0.72	0.37-1.40	0.329			
slow	1.11	0.47-2.61	0.817			
Tumor location						NI
cervical	Ref	Ref	Ref			
thoracic	0.61	0.24-1.54	0.296			
lumbar	1.04	0.42-2.59	0.937			
Number of spinal metastases						NI
single	Ref	Ref	Ref			
multiple	0.97	0.65-1.44	0.880			
BMI (kg/m^2^)						NI
<18.5	1.51	0.50-4.76	0.466			
18.5-30	Ref	Ref	Ref			
>30	1.43	0.42-4.88	0.566			
Surgical procedure						NI
type 1	Ref	Ref	Ref			
type 2	1.18	0.56-2.49	0.672			
type 3	3.70	0.45-3.03	0.223			
Preoperative radiotherapy						NI
yes	Ref	Ref	Ref			
no	0.83	0.55-1.23	0.345			
Preoperative chemotherapy						NI
yes	Ref	Ref	Ref			
no	0.97	0.65-1.44	0.880			
Visceral metastases						
no	Ref	Ref	Ref			
yes	1.39	0.88-2.20	0.163	1.61	0.23-11.14	0.629
Blood loss (liters)	2.00	1.26-3.15	0.003	1.54	0.74-3.21	0.252
Preoperative Frankel score						
A-C	5.56	3.03-11.11	0.001	2.68	1.78-4.04	0.001
D-E	Ref	Ref	Ref			
Blood transfusion						
yes	8.33	4.00-19.23	0.010	3.11	1.61-6.02	0.041
no	Ref	Ref	Ref			
Charlson comorbidity index						
6	Ref	Ref	Ref			
7	3.33	0.72-15.52	0.125	2.01	1.27-3.17	0.013
≥8	5.82	1.36-24.84	0.017	2.29	1.25-4.20	0.017
Operative time (hours)	1.56	1.30-1.87	0.020	1.36	1.14-1.63	0.001

NI, not included.

In the multivariate logistic regression model, four independent risk factors for VTE were further screened out, including preoperative Frankel score (OR=2.68, 95% CI 1.78-4.04, P=0.001), blood transfusion (OR=3.11, 95% CI 1.61-6.02, P=0.041), Charlson comorbidity index (OR=2.01, 95% CI 1.27-3.17, P=0.013; OR=2.29, 95% CI 1.25-4.20, P=0.017), and operative time (OR=1.36, 95% CI 1.14-1.63, P=0.001). The remaining factors (visceral metastases and blood loss) did not show significant statistical significance (**Table 2** and **Figure 1**).

### Establishment and Validation of the Nomogram

**Figure 2:**
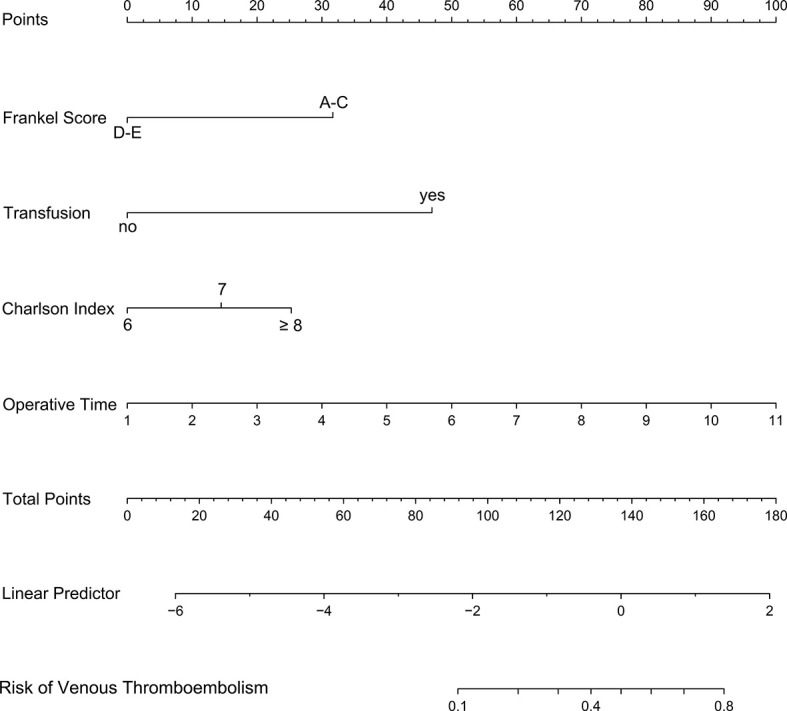
A nomogram model was established using independent risk factors screened out by multivariate regression analysis. The corresponding score for each factor is based on the condition of the patient, which can be determined by making a vertical line upwards (e.g., a patient with blood transfusion will receive between 40 and 50 scores). Add all the scores to get the total score, then find the corresponding point on the total points axis and make a vertical line down to predict the risk of the VTE within 90 days after spinal metastasis surgery.

On the basis of the four independent influencing factors screened out by multivariate logistic regression model, a nomogram prediction model was established (**Figure 2**). Assign these independent influencing factors in the nomogram to line segments of different lengths. The length of line segment represented the weight of the predictive factor. For each independent patient, each influencing factor was scored according to the actual situation, and then the points were added to get a total point. According to the final total point, the estimated risk probability of postoperative VTE for this patient can be obtained. The nomogram showed that the higher the patient’s score, the higher the risk of postoperative VTE.

**Figure 3:**
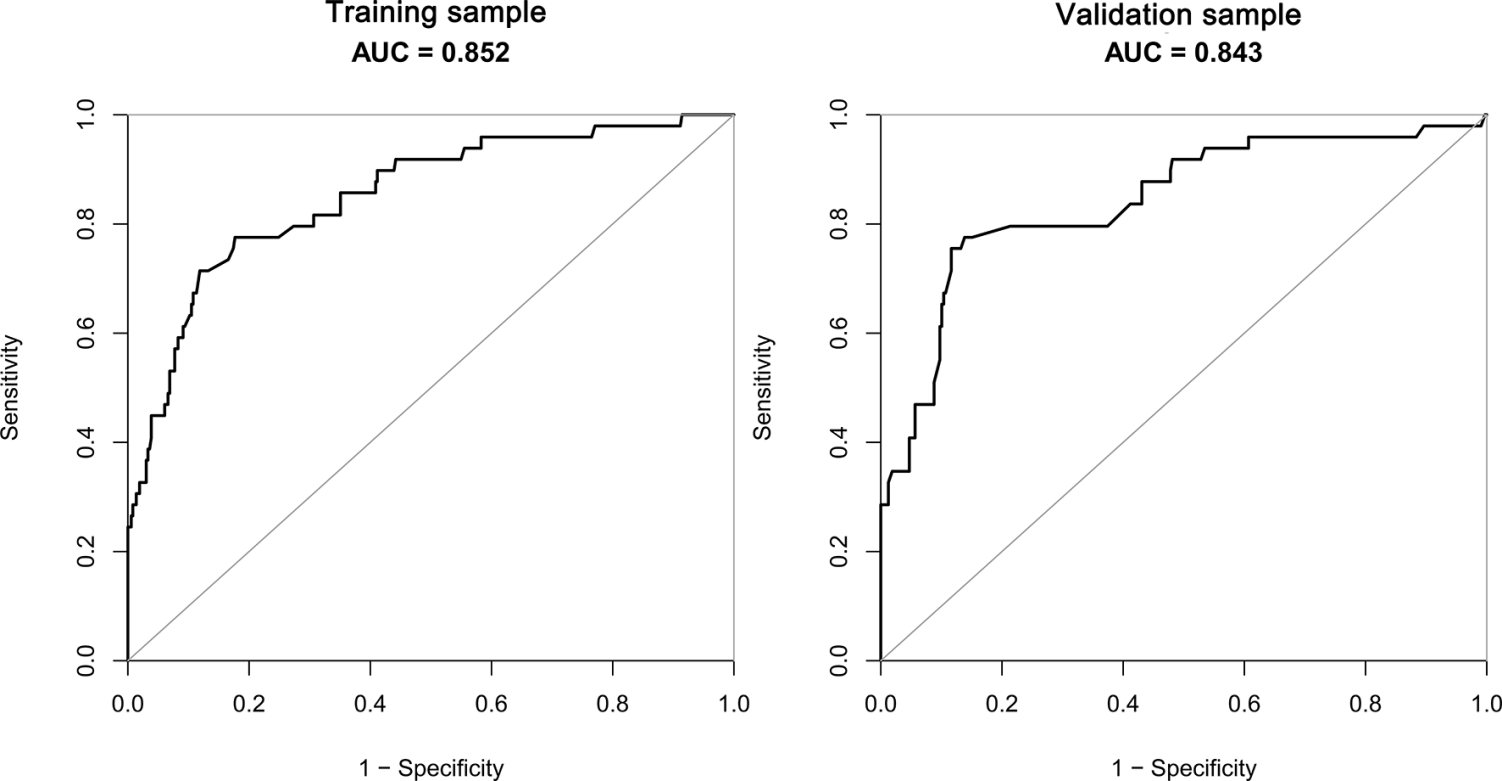
The AUC of training sample (AUC=0.852) and validation sample (AUC=0.843) showed that the model had a high discrimination ability.

**Figure 4:**
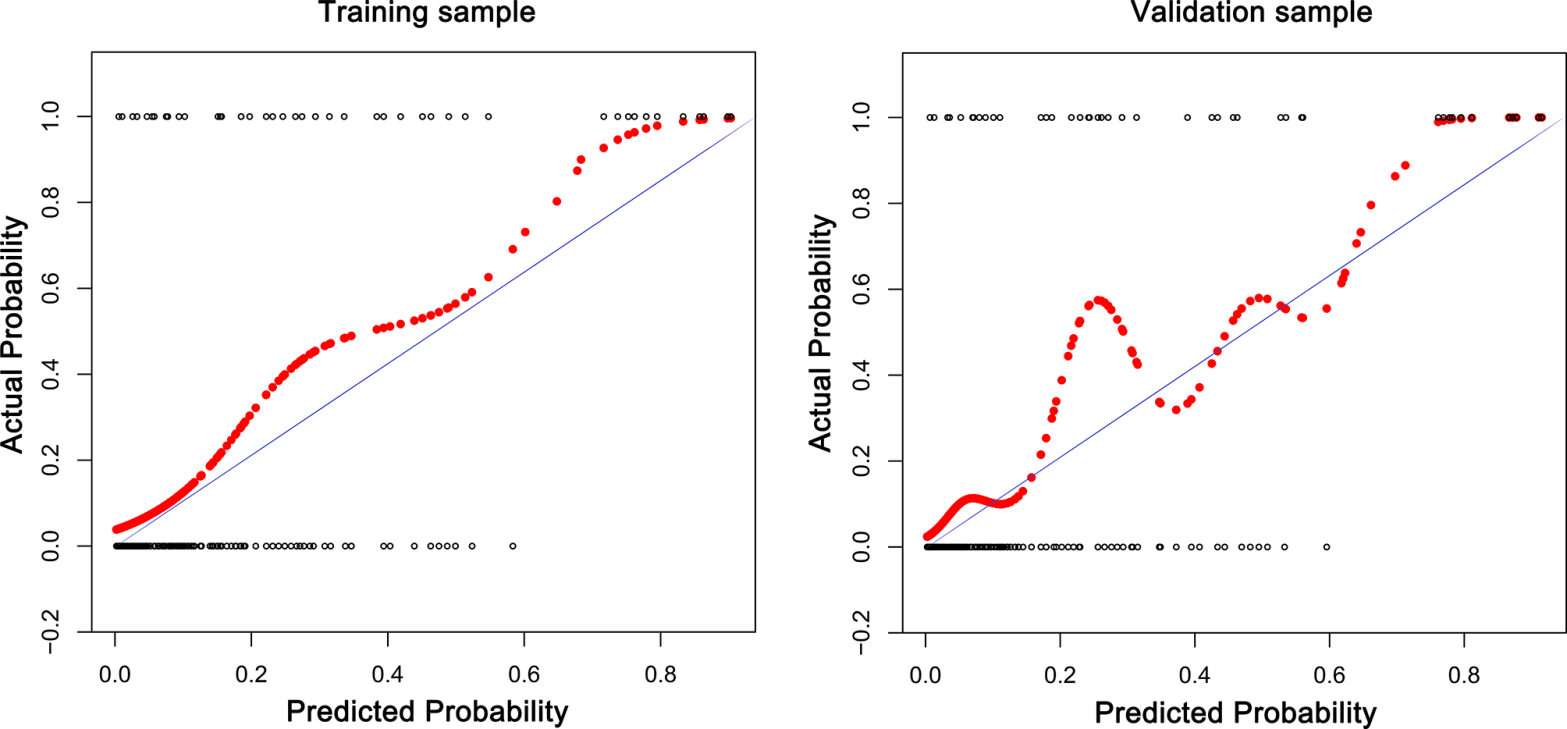
The calibration curves for assessing the consistency between the predicted and the actual risk of postoperative VTE. Favorable consistencies between the predicted and the actual risk evaluation are presented.

The receiver operating characteristic (ROC) curves were drawn in the training sample and the validation sample, and AUC was calculated to determine the discrimination of the prediction model. The results showed that the model had a high discrimination ability (**Figure 3**). The AUCs of the training sample and the validation sample were 0.852 and 0.843, respectively. In addition, the calibration curves were drawn to show the agreement between the predicted value and the true value. Both training sample and validation sample showed that the predicted probability of the nomogram had a strong correlation with the actual situation (**Figure 4**).

## Discussion

As cancer patients live longer and various diagnostic measures continue to improve, the incidence of metastatic spinal disease in the population is increasing. The spine is the third most common site of cancer metastases, second only to the lung and liver (19–21). The treatment of spinal metastases requires multidisciplinary collaboration, including surgery, radiotherapy and chemotherapy. The purposes of surgery are to relieve the symptoms of spinal cord compression, restore and maintain spinal stability, and improve the life expectancy and quality of life of cancer patients as much as possible.

However, there have been several literature proving that cancer and spinal surgery are two major risk factors for postoperative VTE, and VTE is associated with poor prognosis (22–24). Therefore, accurately identifying the risk factors of VTE helps clinicians adjust clinical decisions in a timely manner to adapt to the different conditions of patients. For non-tumor spinal surgery, there have been some studies that discussed the incidence and risk factors of VTE in detail (7, 8, 25), and a predictive score had been established (6). However, for spinal metastasis surgery, the current research results are far from enough. In daily clinical practice, orthopedic oncologists need a predictive model for the postoperative VTE in patients with spinal metastases to assess the patients’ risks and make corresponding interventions. Therefore, the purpose of this study is to screen out independent risk factors for VTE after spinal metastasis surgery and establish a user-friendly predictive model.

The lack of walking function will lead to a high risk of VTE has been proven by several studies. Dermody et al. followed 174 asymptomatic, non-ambulatory neurosurgical patients and found that the incidence of postoperative DVT was 23% (26). Tominaga et al. retrospectively studied the data of patients who underwent spinal surgery and developed postoperative VTE to identify risk factors related to postoperative VTE. Multivariate logistic regression analysis showed that the independent risk factors were preoperative walking disorder and age (7). In patients undergoing surgery for spinal metastases, Zacharia et al. have demonstrated that non-ambulatory status is an independent risk factor for positive finding on preoperative DVT screening. 24% of non-ambulatory patients suffer from DVT, and this incidence of DVT is 4 times higher than that of ambulatory patient population (27). These conclusions indicate that early gait training is important to prevent VTE, although it may take a long time for the muscle strength to reach the level required for walking. The use of robotic suits for neurological rehabilitation may help patients with walking difficulties. Aach et al. reported that the use of hybrid assistive limb exoskeleton can effectively improve the ability to walk on the ground (28).

Blood transfusion is associated with a high risk of VTE in cancer patients (29). One possible explanation may be that a tissue-factor-initiated pathway of coagulation activation on tumor cells appears to trigger coagulation activation in malignancy (30). Regarding the specific components of blood products, most evidence shows that VTE is closely related to red blood cell transfusion, and there is also some evidence that VTE is associated with platelet transfusion (31). In addition, in the study conducted by Kaewborisutsakul and colleagues (9), multivariate regression analysis found that there was a statistical correlation between the infusion of fresh frozen plasma and the high risk of VTE (9). The current study supports the above conclusions, patients who have blood transfusion have three times the risk of suffering from VTE, compared with patients without blood transfusion.

Age-adjusted Charlson Comorbidity Index is a widely used comorbidity scoring system. This score quantifies comorbidities based on the number and severity of the diseases that the patient has endured, and can be used to predict the patient’s risk of death (17). Groot and colleagues introduced the Charlson Comorbidity Index into the study to assess the risk of postoperative VTE in patients with spinal metastases, although the final statistical analysis showed that there was no significant statistical association between the index and the risk of VTE (5). The current research showed that Charlson Comorbidity Index is an independent risk factor for postoperative VTE in patients with spinal metastases, and as the index increases, the risk tends to increase. Certain indicators in the Charlson Comorbidity Index have been proven to be high-risk factors for postoperative VTE, including age (7), diabetes (32), cerebrovascular disease (33), solid tumors (22), and hemiplegia (6, 7). Therefore, we can completely believe that the Charlson Comorbidity Index can predict the arrival of VTE.

Some studies have explored the association between operative time and postoperative VTE. Tominaga et al. found that 20 of 80 patients had VTE after spinal surgery. The median operative time for patients with VTE and without VTE were 212.5 minutes and 177.5 minutes, respectively (7). A large-scale retrospective study showed that longer operative time was independently associated with an increased risk of postoperative symptomatic VTE. The risk of VTE will increase by 15% for every additional hour of surgery (5). They explained that this may require clinicians to consider more measures to prevent symptomatic VTE, such as chemoprophylaxis. Schoenfeld et al. (34) and Piper et al. (6) also determined that operative time > 261 minutes and operative time ≥ 4 hours were independent predictors of VTE after spinal surgery. In the current study, univariate and multivariate regression analyses showed that operative time is an independent prognostic factor affecting postoperative VTE. As the operative time increases by 1 hour, the risk of postoperative VTE will increase by 36%. Our explanation for this phenomenon is: maintaining a supine posture for a long time during the operation will cause part of the venous return to be blocked and blood will be in a hypercoagulable state, which could easily cause blood clots.

In the current study, we are trying to determine the risk factors of VTE after spinal metastasis surgery and further stratify and predict the future condition of patients. This predictive model can help clinicians make evidence-based decisions on when to use chemoprophylaxis, thereby further reducing the incidence of VTE and related medical expenses in patients undergoing spinal metastasis surgery.

There are several limitations in this study. First of all, any retrospective analysis may cause errors due to selection bias and recall bias; however, we reduce the selection bias by expanding the number of hospitals participating in the database construction. Secondly, due to the limitations of the database, we have not been able to analyze the potential predictive value of some indicators for postoperative VTE, such as D-dimer, preoperative hemoglobin, and blood oxygen saturation. Finally, we conducted internal validation, but did not complete external validation, which would have a certain adverse effect on the applicability of the model. Future studies should further evaluate the applicability of this model in other spinal metastasis cohorts and make possible modifications. The external validation can be accomplished by repeating the analyses of various risk factors using data from databases in other countries or regions.

The prediction model for postoperative VTE developed by our team provides clinicians with a simple method that can be used to calculate the VTE risk of patients at the bedside, and can help clinicians make evidence-based judgments on when to use intervention measures. In clinical practice, the simplicity of this predictive model has great practical value. For the pathogenesis and significance of various risk factors of VTE after surgery, further researches are needed.

## Data Availability Statement

The raw data supporting the conclusions of this article will be made available by the authors, without undue reservation.

## Ethics Statement

The studies involving human participants were reviewed and approved by Medical ethics review committee of Tianjin Hospital. The patients/participants provided their written informed consent to participate in this study.

## Author Contributions

H-rZ and M-yX analyzed and interpreted the patient data and were the major contributors in writing this manuscript. R-qQ, Y-cH, and X-gY checked and revised the manuscript. J-kL collected the data and sorted out the material. LY and HZ participated in the conception of the study and manuscript revision. FW, J-yZ, and Y-lZ helped finalizing the manuscript. All authors contributed to the article and approved the submitted version.

## Conflict of Interest

The authors declare that the research was conducted in the absence of any commercial or financial relationships that could be construed as a potential conflict of interest.

## Abbreviations

VTE, venous thromboembolism; DVT, deep vein thrombosis; PE, pulmonary embolism; AUC, area under the curve; ROC, receiver operating characteristic.
